# Inflammatory Bowel Diseases and Gut Microbiota

**DOI:** 10.3390/ijms24043817

**Published:** 2023-02-14

**Authors:** Yuri Haneishi, Yuma Furuya, Mayu Hasegawa, Antonio Picarelli, Mauro Rossi, Junki Miyamoto

**Affiliations:** 1 Department of Applied Biological Science, Graduate School of Agriculture, Tokyo University of Agriculture and Technology, Fuchu-shi 183-8509, Tokyo, Japan; 2 Department of Translational and Precision Medicine, Sapienza University of Rome, 00185 Rome, Italy; 3 Institute of Food Sciences, National Research Council (CNR), Via Roma 64, 83100 Avellino, Italy

**Keywords:** inflammatory bowel disease, gut microbiota, probiotics, fecal microbiota transplantation, immunity, inflammation

## Abstract

Inflammatory bowel disease (IBD) is an inflammatory disease of the gastrointestinal tract, the incidence of which has rapidly increased worldwide, especially in developing and Western countries. Recent research has suggested that genetic factors, the environment, microbiota, and immune responses are involved in the pathogenesis; however, the underlying causes of IBD are unclear. Recently, gut microbiota dysbiosis, especially a decrease in the abundance and diversity of specific genera, has been suggested as a trigger for IBD-initiating events. Improving the gut microbiota and identifying the specific bacterial species in IBD are essential for understanding the pathogenesis and treatment of IBD and autoimmune diseases. Here, we review the different aspects of the role played by gut microbiota in the pathogenesis of IBD and provide a theoretical basis for modulating gut microbiota through probiotics, fecal microbiota transplantation, and microbial metabolites.

## 1. Introduction

The incidence of immune-mediated diseases, such as autoimmune diseases, continues to increase worldwide [1]. Autoimmune diseases are caused by genetic and environmental factors. It is a general term for diseases in which the immune system, instead of eliminating foreign substances, such as bacteria, viruses, and tumors that differ from itself, malfunctions, overreacts, and attacks its own normal cells and tissues. Additionally, the disruption of the intestinal barrier and changes in the intestinal microbiome are thought to be responsible for various autoimmune and metabolic diseases [2]. Irritable bowel syndrome (IBS), inflammatory bowel diseases (IBD), and celiac disease, as well as obesity and diabetes mellitus, play an important pathogenetic role in these alterations [3,4,5]. During diets conducted in the suspicion of these diseases, dysbiosis seems to play an important role in the emergence of increased intestinal permeability (leaky gut). The human intestinal tract contains approximately 1000 species of intestinal bacteria, totaling 100 trillion bacteria, which are likely to be involved in regulating host homeostasis [6,7]. In recent years, a comprehensive analysis of intestinal bacteria has actively identified gut microbiota species and their metabolites that are directly involved in the pathogenesis of metabolic diseases. These are expected to play an important role in maintaining host homeostasis [8,9,10,11]. Clearly, the gut microbiota influences the pathogenesis and pathology of diseases [12,13,14], and the elucidation of the gut microbiota may contribute to reducing disease risk (Table 1).

IBD is an autoimmune disease. Its incidence continues to rapidly increase worldwide, especially in developing and Western countries [22]. IBD is an inflammatory disease that results from immune disorders arising from complex interactions between genetic and environmental factors such as diet, alcohol, stress, insufficient sleep, and the microbiome [23]. IBD is characterized by the chronic relapse and remission of inflammation in the gastrointestinal tract. It is classified into two main types: ulcerative colitis (UC) and Crohn’s disease (CD). The quality of life in IBD patients has decreased because of symptoms such as diarrhea, abdominal cramps, hematochezia, weight loss, fever, fatigue, and anemia [24]. CD is characterized by transmural inflammation affecting any part of the gastrointestinal tract such as the mouth, anus, and entire intestinal layer, whereas UC typically affects the mucosal layer of the colon [25]. As the species comprising the gut microbiota and the composition vary in a different part of the gastrointestinal tract [26], the impact of UC and CD might be in different areas of inflammation with distinctive intestinal microbiota, which may develop and aggravate the pathological condition. Unlike infectious diseases, IBD is caused by defects in the autoimmune system in the gastrointestinal tract, and a treatment that can completely cure IBD is still lacking [27]. The disruption of the immune system caused by dysbiosis of the gut microbiota increases inflammation in the gastrointestinal tract and leads to the onset of IBD [28]. Therefore, improving the gut microbiota and identifying the specific bacterial species in IBD could be significant for understanding the pathogenesis and treatment of IBD and autoimmune diseases.

Here, we aimed to provide an overview of the interaction between IBD and the gut microbiota, especially the factors, prevention, and therapy for IBD. We have also reviewed the gut microbiota that are associated with IBD and modulate the microbiome using probiotics, fecal microbiota transplantation (FMT), and microbial metabolites.

## 2. Gut Microbiota and IBD

Currently, in Europe and the United States, 3.6 million people suffer from IBD, including UC and CD, which are becoming increasingly prevalent worldwide [29]. IBD is an autoimmune disease involving intestinal inflammation; however, it has garnered attention due to the crosstalk between the gut microbiota and autoimmune systems. The gut microbiota has been shown to trigger IBD-initiating events. The composition of the gut microbiota is different between healthy participants and IBD patients [30]. The use of antibiotics also leads to the onset of IBD due to the alteration of the composition and function of the gut microbiota [31]. The interaction between the gut microbiota and host plays a critical role in maintaining the function of the host’s immune system [32]. Changing environmental events such as the overuse of antibiotics and changes in diet may enhance autoimmune and inflammatory disorders by modulating the gut microbiota composition, leading to dysbiosis [33]. Thus, a crucial relationship exists between IBD and the microbial communities in the human gut. Although IBD is widely attributed to altered interactions between gut microbes and the intestinal immune system, the exact mechanism underlying gut microbiota dysfunction in IBD remains unclear [34]. Although the pathogenesis of IBD is unknown, the inflamed gastrointestinal tract in patients with IBD is a common feature of an imbalance (dysbiosis) in the gut microbiota. Recently, much evidence has been provided to show that gut dysbiosis leads to the disruption of immune tolerance, which may induce or exaggerate IBD [35].

Studies with human participants have clarified that the composition of the gut microbiota is different in patients with IBD compared with that in healthy subjects [30]. Additionally, the gut microbiota is different between patients with UC and CD [36]. Analyses of the gut microbiota in patients with IBD worldwide has led to the observation that dysbiosis involves an increase or decrease in specific intestinal bacterial species in patients with IBD. Morgan et al. reported that a large, long-term prospective cohort study showed that patients with IBD have characteristic gut microbiota compared with those in healthy participants [37]. The abundance of *Roseburia* and *Phascolarctobacterium* was significantly decreased, whereas that of *Clostridium* was increased in the gut microbiota of patients with UC or CD. *Roseburia* is associated with anti-inflammatory regulatory T cell production in the intestinal tract [38], and *Phascolarctobacterium* consumes only succinate and produces propionic acid when co-cultured with *Paraprevotella* [39]. Propionic acid is a short-chain fatty acid (SCFA) that has anti-inflammatory effects [40]. Patients with IBD have decreased SCFA-producing *Phascolarctobacterium*, suggesting that the anti-inflammatory effects of SCFAs might be reduced, which could consequently exacerbate IBD symptoms.

The diversity and species richness of the *Clostridium leptum* group, which is one of the major bacterial groups comprising 16–25% of the human gut microbiota, were different between healthy participants and those with IBD in the remission phase [41]. The *C. leptum* group, also called Clostridial cluster IV, includes *Faecalibacterium prausnitzii*, *Eubacterium*, and *Ruminococcus*. *F. prausnitzii* shows a reduced abundance in patients with CD and in mouse models of colitis [42]. The *C. leptum* group dominated by *F. prausnitzii* showed a significantly decreased abundance in the fecal microbiota of patients with CD or UC and was detected during both the disease activity and remission stages. This suggests either that the reduction in *F. prausnitzii* levels leads to the onset of IBD or that IBD onset events may have caused the decrease in *F. prausnitzii* levels [41]. Additionally, the study showed that a low abundance of *F. prausnitzii* in the ileum of patients with IBD during surgical resection is a risk factor for endoscopic recurrence. Patients with CD who were treated for endoscopic recurrence at 6 months showed lower levels of Firmicutes, especially those of *F. prausnitzii*, which is the main bacteria of the *C. leptum* group. Moreover, *F. prausnitzii*—a defective commensal bacterium in CD—was found to exert anti-inflammatory effects in vitro and in vivo. This suggests that the use of *F. prausnitzii* as a probiotic to improve gut dysbiosis leads to the remission of serious CD symptoms [42].

**Figure 1:**
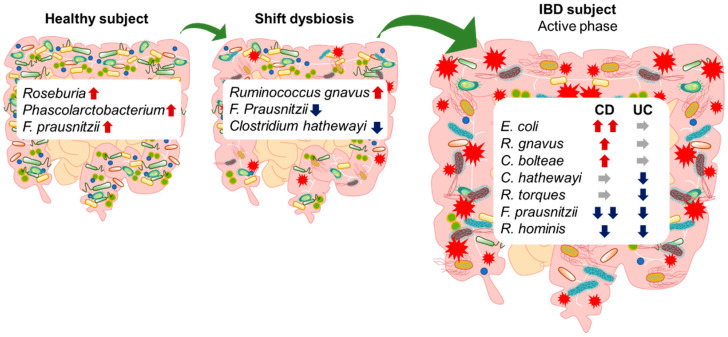
The gut microbiota is altered in IBD patients compared to healthy subjects. Up-arrows indicate an increase, down-arrows indicate a decrease, and horizon-arrows indicate no changes.

Generally, close relatives living in the same environment have similar gut microbiota, but a study in twins reported that when one twin pair was healthy and the other had IBD, the microbial compositions in the twins were different [43]. As this study included twins, it eliminated the influence of the genetic background on the abundance of *F. prausnitzii* and other specific genera such as *Alistipes*, *Collinsella*, and *Ruminococcaceae*. This result suggests that the onset of IBD might be more strongly influenced by environmental factors than by genetic factors, especially with regard to the gut microbiota, which were reduced in IBD patients compared to healthy subjects. These results suggested that the onset of IBD might be more strongly influenced by environmental factors, especially gut microbiota, than by genetic factors. Moreover, a multi-omics analysis including metagenomic, metatranscriptomic, metabolomic, and other analyses revealed that the gut microbiota and their metabolites in CD and UC were different during the active and remission phases [44]. Thus, a multi-omics approach identified the characteristics of gut microbiota in IBD and the metabolites produced by the gut microbiota. This may clarify the impact of the crosstalk between the gut microbiota, their metabolites, and the host on the pathogenesis of IBD. Furthermore, follow-up studies that characterize the microbial features in several hosts could provide treatment plans adapted to each individual. However, a larger sample size may be needed. Nevertheless, these molecular biological approaches could be helpful in the development of novel therapies targeting host–microbe interactions as predictive biomarkers of IBD onset, progression, and recovery (Figure 1).

## 3. Effect of Probiotics, Prebiotics, and Symbiotics on IBD

Despite the lack of definitive therapies for IBD, approaches targeting gut microbiota have been reported in recent years to cure or improve IBD. VSL#3 is one of the most well-known probiotics for IBD treatment. VSL#3 is a probiotic mixture that includes the following eight bacteria at a concentration of 450 billion live bacteria per sachet: *Bifidobacterium breve*, *B. longum*, *B. infantis*, *Lactobacillus acidophilus*, *L. plantarum*, *L. paracasei*, *L. delbrueckii subsp. Bulgaricus*, and *Streptococcus thermophilus* [45]. VSL#3 improves IBD symptoms by improving the intestinal environment, enhancing microbial diversity, and modulating the concentration of specific bacteria such as increasing that of *Bifidobacterium* and decreasing that of *Turicibacter* in animal models [46,47,48]. Moreover, the administration of VSL#3 improved inflammatory responses in the intestine, suggesting that the remodeling of the composition of the gut microbiota by VSL#3 may have suppressed intestinal inflammation, which ameliorated IBD pathologies. Additionally, several clinical studies have reported that VSL#3 is effective in the improvement of IBD [49,50,51,52]. Although the safety of VSL#3 is recognized [53], the issues surrounding the use of VSL#3 are yet to be elucidated in clinical applications; therefore, the confirmation of the efficacy of VSL#3 could lead to a widely spread use of VSL#3 for IBD treatment.

**Figure 2:**
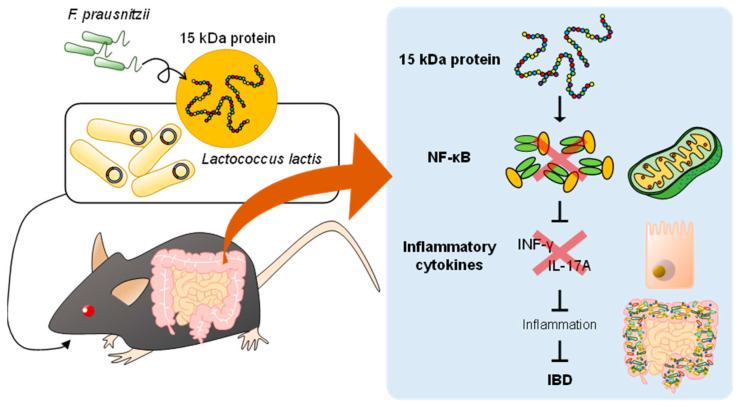
Improved intestinal environment alleviates IBD. Modulating gut microbiota, such as probiotics, fecal microbiota transplantation (FMT), and gut microbial metabolites, can alleviate IBD symptoms by suppressing gastrointestinal inflammation.

*F. prausnitzii* decreases pro-inflammatory cytokine levels and promotes the secretion of anti-inflammatory cytokines, suggesting that *F. prausnitzii* may have the ability to regulate immunity [42]. However, whether the bacterium itself or the metabolites it produced exerted the anti-inflammatory effect is unclear. However, some studies have revealed that the metabolites produced by *F. prausnitzii* exert anti-inflammatory effects in CD [54]. An unknown bioactive peptide derived from a 15 kDa protein (ZP05614546.1), which has anti-inflammatory effects, was discovered by screening metabolites from *F. prausnitzii*. Quévrain et al. also found that metabolites produced by *F. prausnitzii* exerted anti-inflammatory effects by the inhibition of NF-κB signaling in vitro and in vivo and that these metabolites significantly reduced NF-κB pathway activation in a dose-dependent manner. *Lactococcus lactis* transfected with plasmids encoding anti-inflammatory peptides produced by *F. prausnitzii* alleviated dinitrobenzene sulfonic acid (DNBS)-induced colitis in mice [54] (Figure 2).

Several reports have indicated that a certain probiotic bacterial strain improved IBD or colitis. *Escherichia coli Nissle* 1917 (EcN) has been proposed as an efficient probiotic for UC in a clinical trial [55]. In a double-blind, double-dummy study, patients with UC in remission received a bacterial preparation containing viable EcN or mesalazine in tablet form for 5 days. The formulation was meant to prevent UC relapse. After a 12-month follow-up, no difference was observed in the relapse of UC between the EcN-treated and mesalazine-treated groups. Hence, EcN could be a new probiotic formulation to replace mesalazine. Some *Lactobacillus* species are useful for the treatment of IBD. The oral administration of *L. rhamnosus* GG reduced inflammatory cytokine levels and improved colonic histology scores, which prevented colitis relapse in rats during antibiotic treatment [56]. Additionally, a study using mouse models of colitis showed that *Enterobacter ludwigii* was effective in alleviating the symptoms of colitis [57]. In a dextran sulfate sodium (DSS)-induced colitis mouse model, compared with that of other antibiotic treatments, metronidazole had the best effect in reducing colitis, which was related to an increase in the abundance of the gut microbiota species *E. ludwigii*. The administration of *E. ludwigii* induced Treg differentiation via metabolites from *E. ludwigii* and increased the immune tolerance response, which reduced the susceptibility to DSS-induced colitis in mice [57].

Prebiotics are non-digestible food ingredients that exert beneficial effects by selectively stimulating the growth of bacteria that can improve host metabolism in the gastrointestinal tract. The abundance of *Bifidobacteria* increased in patients with CD following prebiotic oligofructose and inulin ingestion [58]. The improvement in the intestinal environment through prebiotic administration is well documented in not only animal models but also humans. Therefore, these results suggest that prebiotics may be effective food ingredients for improving the intestinal environment in IBD pathology. Symbiotics are microbial therapeutics that, when administered in combination with prebiotics and probiotics, can benefit the host health to a greater degree than prebiotics or probiotics alone. In the meta-analysis, the administration of probiotics, prebiotics, or symbiotics to patients with IBD alleviated or improved IBD symptoms in all groups and increased the abundance of beneficial *Bifidobacteria* [59]. In particular, the symbiotic group showed the highest improvement, indicating that symbiotics may be more effective than prebiotics or probiotics in improving the pathophysiology of IBD.

## 4. FMT for IBD

FMT is an emerging therapy that may be a potential therapeutic approach for IBD. Based on the key role of gut microbiota in the pathogenesis of IBD, FMT can restore intestinal mucosal immune homeostasis in patients with IBD, which is a current research hotspot. FMT can be used to treat moderate to severe IBD complicated by recurrent or refractory *Clostridium difficile* infection [60,61,62,63].

FMT may be an efficient therapy for IBD in animal models. In mice with DSS-induced colitis, FMT suppressed colon inflammation and restored intestinal barrier functions and the amelioration of colitis by suppressing colon damage and recovering colon length [64,65]. Moreover, FMT-treated mice showed a decrease in the abundance of specific gut microbiota, such as *Bacteroides acidifaciens*, *Escherichia-Shigella*, and *Blautia*, which induce inflammation. This suggests that FMT mitigated colitis in mice by suppressing colitis-associated inflammation by improving the intestinal microbiota. Zhang et al. reported that FMT, in addition to improving colitis pathogenesis in mice with gut dysbiosis, also significantly increased the intestinal bacterial metabolite SCFA levels [66]. SCFAs regulate inflammatory responses and restore immune function [67], and Zhang et al. demonstrated that FMT may alleviate IBD by altering the composition of gut microbiota by increasing the SCFAs produced by gut microbiota. FMT is currently being investigated in registered and ethical clinical research studies and has not been approved for clinical application. Further research is needed to better understand the mechanisms underlying the effects of FMT to develop more effective regimens for treating IBD [68,69,70].

A double-blind study in patients with UC investigated the association between the remission of UC symptoms and gut microbiota [71]. Following treatment with FMT or a placebo for active UC, the gut microbiota was altered in patients in the FMT group. Additionally, increased α- and β-diversity of the gut microbiota and an increased abundance of *Eubacterium hallii*, *Roseburia inulivorans*, *Eggerthella*, and *Ruminococcus bromii* were observed in patients with UC remission in the FMT group. Moreover, increased levels of gut microbial metabolites (such as SCFAs and secondary bile acids) in feces were observed in patients with UC remission in the FMT group [72]. Furthermore, the treatment of FMT in patients with active CD also showed improvements in symptoms. Similarly, an increase in α-diversity was observed in patients with CD remission. Therefore, the FMT-induced alteration of the gut microbial composition may at least partially alleviate the signs and symptoms of IBD.

## 5. Microbial Metabolites and IBD

The Westernization of diets, such as an increased prevalence of high-fat and high-sugar diets, has induced both non-communicable diseases such as obesity, diabetes, and cardiovascular diseases and immune-related diseases such as IBD, hay fever, and celiac disease [33,73]. As diet could have a significant influence on the host immune system, developing methods for preventing and ameliorating autoimmune diseases using diet therapy is essential [74,75,76]. Celiac disease is another autoimmune disease that causes inflammation of the intestinal tract similar to IBD, but celiac disease symptoms are dramatically alleviated by the removal of gluten contained in wheat from the diet [77]. IBD does not have an established diet therapy; however, in recent years, the quantity and quality of the diet has been suggested to alter the host’s intestinal microbiota and influence the development of IBD [78]. For example, food emulsifiers, which are widely contained in processed foods in Western diets, increase bacterial intestinal permeability in vitro and promote early lesions of inflammation in IBD, whereas dietary fiber, which is scarce in Western diets, inhibits these responses [79]. The administration of food emulsifiers to mice promoted obesity and inflammation and exacerbated the symptoms of colitis. In addition, the gut microbiota composition was dramatically altered in mice treated with food emulsifiers compared with that in the controls [80]. Additionally, the quantity of SCFAs and bile acids produced by gut microbiota was altered in emulsifier-supplemented mice, indicating that emulsifiers may affect the gut microbiota and its metabolites, which may induce obesity and colitis [80].

SCFAs are fatty acids with two to six carbon atoms. The major SCFAs are acetic, propionic, and butyric acid. SCFAs are major intestinal bacterial metabolites produced by the fermentation of non-digestible polysaccharides, such as dietary fiber, by intestinal bacteria. SCFAs contribute to biological homeostasis by being used as an energy source for colonic epithelial cells and fatty acid synthesis in peripheral tissues [81]. SCFAs play an important role in host metabolic regulation and may contribute to improving the amelioration of immune disorders such as IBD [82]. SCFAs were decreased in the feces of patients with IBD compared with that of healthy subjects, which might be attributed to dietary differences [83]. The SCFA levels in the fecal samples of patients with UC were significantly lower compared with those of healthy participants, but the SCFA levels increased during the remission of UC, indicating that SCFAs could change with the stage of disease activity [84]. Butyrate derived from commensal gut microbes effectively facilitated anti-inflammatory M2 macrophage polarization, which influenced the immune system and improved intestinal inflammation in a mouse DSS-induced colitis model. Thus, SCFAs from gut microbiota may be a novel activator with anti-inflammatory properties and a therapeutic target for IBD [85]. The administration of *Pediococcus pentosaceus* LI05 changed gut microbial profiles by promoting SCFA production, modulating the gut microbial composition, and increasing the abundance of specific genera (*Akkermansia*, *Faecalibacterium*, and *Ruminiclostridium_5*), which ameliorated DSS-induced colitis [86]. Moreover, the ingestion of β-glucans, which are non-digestible complex dietary polysaccharides commonly contained in barley, increased SCFA levels, which also ameliorated DSS-induced colitis [87]. Mice fed saturated fatty acids, which are abundant in dairy products, meat, and palm oil, showed increased concentrations of the sulfite-reducing pathogen *Bilophila wadsworthia* hydrogen sulfide and secondary bile acids produced by *B. wadsworthia*, which promoted the onset of colitis [88]. In a clinical study, the short-term consumption of an animal-based diet significantly increased the *B. wadsworthia* abundance and the expression level of gut bacterial genes encoding bile acid and sulfite reductase, suggesting that dietary fat is abundant in the Western diet and may be capable of triggering IBD by altering the intestinal environment [89].

The gut microbiota metabolizes primary bile acids synthesized from cholesterol in the liver into secondary bile acids. Recently, it has been reported that the number of secondary bile acids in plasma and feces decreases in patients with IBD [90]. The administration of human microbiota decreases the production of secondary bile acids in donor mice, which may disrupt the immune response and induce IBD onset [91]. Tryptophan, an essential amino acid, is converted by the gut microbiota into quinolinic acid, which has been associated with IBD [92]. Quinolinic acid was significantly increased in patients with IBD, and gut microbial enzymes involved in the tryptophan metabolism were activated in a large cohort study of 535 IBD patients in Germany, including patients with UC and CD. This result suggests that gut dysbiosis may induce IBD by promoting the conversion of tryptophan to quinolinic acid. Aryl hydrocarbon receptor (AhR) ligands include well-known chemicals such as dioxin and benzopyrene. AhR ligands exert anti-inflammatory effects by regulating various immunological responses, and, recently, it has been shown that the administration of AhR ligands improves gastrointestinal symptoms in patients with UC [93]. These results suggest that diet-induced changes in the gut microbiota and their metabolites alter the host immune system and metabolism, which may contribute to the development of IBD. However, the immune mechanism underlying the role of the metabolites in IBD remains unclear. Therefore, understanding the interaction between food, gut microbiota, and bacterial metabolites may play a key role in diet therapy for IBD.

## 6. A Potential Innovative Strategy by Postbiotic Treatment

Impairments in IBD immune regulation have been described at different levels. Regarding the lymphocytic response, CD has been mainly associated with a Th (T helper)1/Th17 condition, whereas UC is characterized by an altered Th2 response [94]. An association of dysbiosis in IBD pathogenesis is also evident. Consequently, the administration of probiotics and their derivatives might be considered for reducing the inflammation or even strengthening the intestinal epithelial barrier [95]. Postbiotics mainly refer to biologically active components secreted by bacteria [96]. Their advantages over probiotics include a reduced risk of infection or potential side effects triggered by the administration of viable microorganisms to immunocompromised individuals. Among postbiotics, self-assembling protein subunits surface-layer proteins (Slp) have been recently considered. Slp constitute the first line of contact and are directly involved in several molecular mechanisms responsible for beneficial health effects [97]. Slp subunits are held together and are attached to cell wall carbohydrates by non-covalent interactions [98]. Moreover, Slp are associated with additional cell surface proteins, named S-layer-associated proteins (SLAP). These proteins exhibit immunomodulatory abilities as well as other biological functions [99]. Slp and phagocytic cells expressing sensing receptors synergistically interact to fine-tune the T cell signaling that is critical for protecting the host against pathogenic inflammation [100]. Pretreatment with Slp from *Lactobacillus acidophilus* NCFM reduced proinflammatory cytokines [101]. In vivo, SlpA from *L. acidophilus* NCFM reduced intestinal inflammatory cytokines and TLR4 and COX2 expression in a DSS-induced colitis mouse model [102]. More recently, a protective role of glycosylated SlpA expressed by the Propionibacterium strain P. UF1 in chemically induced colitis has been shown [103]. Histological analysis demonstrated severe crypt loss with associated mucosal and submucosal inflammation in the colonic tissues of mice gavaged with PBS or ΔlpsA P. UF1, while DSS-induced colitis was substantially mitigated in mice gavaged with wild type P. UF1. In vitro results suggested that glycosylated LpsA regulated colonic DC metabolic function during intestinal inflammation. In particular, the glycosylation of LpsA was shown to regulate the interaction with the sensing receptor SIGNR1. Activating critical signals involving SIGNR1 and its human homolog, DC-SIGN, may have relevance for developing therapeutic strategies for mitigating intestinal inflammation. Taken together, these reported preclinical studies are pivotal for a deeper understanding of beneficial Slp and pave the way for the development of new postbiotic therapeutic strategies for potentially treating IBD.

Biomarkers targeting the gut microbiota represent noninvasive and reasonable methods that may enable the early detection of disease, the prognosis of the disease course, and even personalized treatments for individual pathogenesis. Many scientists have evaluated biomarkers for IBD targeting the gut microbiota in recent years [104,105,106]. *Klebsiella oxytoca*, *Morganella morganii*, and *Citrobacter amalonaticus* increased in patients with CD, and *C. portucalensis*, *C. pasteurii*, *C. werkmanii*, and *Proteus hauseri* increased in patients with UC, indicating that specific gut microbial species can be used to distinguish the pathologies of CD and UC [107]. Additional research on the species of gut microbiota that are associated with the onset, pathogenesis, or pathophysiology of IBD will help in diagnosing IBD, as well as determining its prognosis and developing treatment options.

## 7. Conclusions

A massive effort in recent years to elucidate microbiome–IBD interactions has led to a better understanding of the pathophysiology of IBD. Patients with IBD and colitis model mice showed altered gut microbial profiles compared with those of healthy participants and mice, suggesting that treatment with probiotics and FMT may ameliorate IBD symptoms by targeting the gut microbiota. Moreover, since dysbiosis of the gut microbiota might result from changes in the diet, qualitative differences in the diet could be expected to restore the intestinal microbiota in patients with IBD and contribute to the improvement of their pathophysiology. Therefore, the quality of the daily diet contributes to improving the gut microbiota and may ameliorate the pathogenesis of IBD.

## Figures and Tables

**Table 1:** Autoimmune diseases and gut microbiota.

Diseases	Gut Microbiota	References
Type 1 diabetes	*Lactobacillaceae* ↓ *Rikenellaceae* ↓ *Porphoromadaceae* ↓	[15]
	*Ruminococcaceae* ↓ *Ruminococcus* ↓	[16]
Multiple sclerosis	*Lactobacillaceae* ↓ *Bacteroidaceae* ↓ *Prevotellaceae* ↓	[17]
	*Akkermansia* ↑	[18,19]
	*Erysipelotrichaceae* ↑ *Lactobacillus reuteri* ↑	
Rheumatoid arthritis	*Collinsella* ↑ *Faecalibacterium* ↑ *Eggerthella* ↑	[20]
Celiac disease	*Dialister invisus* ↑ *Parabacteroides* ↑ *Lachnospiraceae bacterium* ↑	[21]

↓; indicates the decrease. ↑; indicates the increase.

## Data Availability

Not applicable.
